# Divergent patterns of probabilistic reasoning in humans and GPT-5

**DOI:** 10.3389/fpsyg.2026.1782184

**Published:** 2026-03-03

**Authors:** Pegah Imannezhad, Emmanuel M. Pothos, Andy J. Wills

**Affiliations:** 1 Department of Psychology, City St George’s, University of London, London, United Kingdom; 2 School of Psychology, University of Plymouth, Plymouth, United Kingdom

**Keywords:** AI participants (AI subjects), complementarity, conjunction fallacy, disjunction fallacy, GPt-5, human vs. AI cognition, large language models (LLMs), probabilistic reasoning

## Abstract

Large Language Models (LLMs) such as GPT‑5 are increasingly consulted for advice across a wide range of domains, yet little is known about how their probability judgments compare to those of humans. This study examined GPT‑5’s adherence to classical probability rules, focusing on conjunction fallacies, disjunction fallacies, and violations of binary complementarity. Using a large dataset on human probabilistic judgments, in which participants displayed multiple types of fallacies, we tested GPT‑5 on the same task and with matched participant profiles. GPT‑5 produced only single conjunction or disjunction fallacies and showed near‑perfect compliance with binary complementarity constraints. Its overall response pattern aligned with predictions of early quantum‑probabilistic models rather than more recent variants incorporating noise. These findings suggest that GPT‑5 implements a more coherent and internally consistent form of probabilistic reasoning compared to naïve human participants.

## 1 Introduction

Large Language Models (LLMs) have rapidly transformed natural language processing, driving breakthroughs in text generation, translation, summarizing, and interactive artificial intelligence (AI) systems (Bommasani et al., 2021; Brown et al., 2020). Their integration into academic, professional, and public settings has fuelled both optimism and concern regarding their growing influence on cognitive work, decision-making, and epistemic reliance on automated systems (Bommasani et al., 2021; Weidinger et al., 2021). As LLMs increasingly function as partners in judgment and reasoning tasks, understanding their strengths and limitations in probabilistic inference becomes critical.

Recent model generations, including OpenAI’s GPT-5, are explicitly optimized for complex multi-step reasoning and knowledge-intensive decision support (OpenAI, 2025). Such systems may therefore be perceived as credible sources of consultation on probabilistic tasks, given (i) their access to large bodies of human-generated knowledge, (ii) their status as cutting-edge AI, and (iii) their frictionless accessibility without social scrutiny (of course, there are concerns regarding data privacy, though related issues are typically poorly understood at best). Real-world usage patterns already point to this shift: students seek help with academic reasoning, professionals turn to LLMs for second opinions on uncertain tasks, and some individuals consult them for personal guidance (Bright et al., 2024; Hou et al., 2024; Jung et al., 2024). Yet the quality of advice supplied by LLMs remains insufficiently examined. Their effectiveness and trustworthiness ultimately depends on two dimensions: (1) accuracy of domain-relevant knowledge and (2) adherence to normative standards of rational decision-making. The first dimension is highly contextual and task-specific, whereas the second relates to a broader debate in cognitive science: to what extent are probabilistic inferences normatively rational, and to what extent are they systematically biased (Kahneman and Tversky, 1979; Tversky and Kahneman, 1983)?

Normative probabilistic inference is grounded on the principles of Bayesian probability theory. Under this framework, probabilistic judgments are expected to satisfy fundamental constraints such as additivity, complementarity, and overall coherence. These requirements have played a central role in both cognitive science and formal probabilistic modelling (Griffiths et al., 2008; Oaksford and Chater, 2009; Tenenbaum et al., 2011). Decades of behavioural research reveal that human probabilistic reasoning is often consistent with Bayesian principles, but also invariably systematically deviates from these principles, giving rise to robust evidence for cognitive biases such as the conjunction and disjunction fallacies (Carlson and Yates, 1989; Kahneman and Tversky, 1979; Tversky and Kahneman, 1983).

### 1.1 Classical probabilistic reasoning fallacies

A central foundation of the present study concerns several well-documented departures from normative probability theory in human judgment, the conjunction fallacy, the disjunction fallacy, and violations of binary complementarity. The first two biases represent some of the most reliable phenomena in the psychology of judgment and decision-making, having been repeatedly observed across diverse populations, measurement approaches, and experimental paradigms (Dulany and Hilton, 1991; Fisk, 2002; Moro, 2009; Sides et al., 2002; Tversky and Koehler, 1994). Violations of binary complementarity were first systematically reported in Huang et al. (2025) and, though their robustness across paradigms has yet to be established, they are included here because they are particularly surprising (arguably more so than the more common conjunction and disjunction fallacies).

*Conjunction fallacy*: the conjunction fallacy is one of the most influential and extensively replicated violations of Bayesian theory. In their seminal work, Tversky and Kahneman (1983) demonstrated that individuals often judge the probability of a conjunction of two events to exceed that of one of its constituent events. Participants read a description of Linda, designed to match the stereotype of a feminist and were then asked to assess the likelihood of several statements about her. Respondents consistently rated the statement “Linda is a feminist and a bank teller” as more probable than “Linda is a bank teller”, despite the logical impossibility of a conjunction exceeding the probability of either component (Kolmogorov, 1950).

This reasoning error directly violates the conjunction rule, which states that for any events A and B (Equation 1):


P(A∧B)≤P(A),P(A∧B)≤P(B) (1)

The fallacy persists across variations intended to clarify task interpretation, reduce pragmatic confounds, and mitigate stereotype-driven responses, underscoring its robustness and theoretical importance (Dulany and Hilton, 1991; Moro, 2009; Tentori et al., 2004).

Conjunction fallacies can be classified into single versus double variations. A single violation occurs when the conjunction is judged more probable than one marginal event [e.g., P(A∧B)>P(A) ], whereas a double violation occurs when it is judged more probable than both A and B. Double violations represent a stronger form of probabilistic incoherence and are frequently interpreted as reflecting particularly strong reliance on heuristics, such as representativeness, judgment strategies (Crupi et al., 2018; Wojciechowski and Pothos, 2018; Yates and Carlson, 1986).

*Disjunction fallacy*: the disjunction fallacy represents a violation complementary to the conjunction fallacy: individuals judge the probability of a disjunction (one possibility *or* another possibility to be true) to be lower than the probability of one of its constituent events. This contradicts another fundamental axiom of Bayesian theory (Kolmogorov, 1950), which states that the likelihood of a disjunction cannot be less than that of either component event, as shown in Equation (2):


P(A∨B)≥P(A),P(A∨B)≥P(B) (2)

Nevertheless, empirical research demonstrates that such violations do occur in human judgment (Carlson and Yates, 1989). Double disjunction fallacies have also been documented—cases in which the disjunction is rated as less probable than both constituent events simultaneously (Huang et al., 2025).

*Binary complementarity violations*: a foundational axiom of classical probability theory is binary complementarity, which states that the probabilities of an event and its negation must sum to unity (Kolmogorov, 1950), as shown in Equation (3):


P(A)+P(¬A)=1 (3)

When extended to joint probabilities, complementarity entails that the exhaustive set of possibilities must also sum to unity, as shown in Equation (4):


P(A∧B)+P(A∧¬B)+P(¬A∧B)+P(¬A∧¬B)=1 (4)

Although complementarity may appear trivial—an immediate consequence of the axioms of probability—empirical evidence, however, indicates that complementarity is not universally respected in human judgment. Research in subjective probability elicitation shows that individuals generally approximate complementary coherence when negations are explicitly stated and tasks are transparent (Budescu et al., 1997; Tversky and Koehler, 1994; Wallsten et al., 1993). Violations emerge, however, in contexts where cognitive interpretation, task structure, or framing introduce ambiguity. For example, complementarity failures have been documented in choice behaviour (Macchi et al., 1999; Shafir, 1993), similarity-based categorization, where perceived overlap between concepts distorts binary relations (Tversky and Gati, 1978), and in framing tasks in which negations are implicit rather than explicit (Epping and Busemeyer, 2023). The most systematic evidence for violations of binary complementarity has been reported in Huang et al. (2025). In their work, even though events and their negations were not explicitly represented (i.e., participants did not see A and not A), the corresponding judgments for marginals were in the same block and so participants violated this constraint despite essentially having these judgments side by side—this is a very surprising finding. Violations of binary complementarity were also reported on conjunctions in the rest of their experiment.

While complementarity violations are less frequently discussed than conjunction or disjunction fallacies, they highlight important constraints in human probabilistic reasoning and provide a valuable benchmark for evaluating coherence in artificial systems such as LLMs.

Summing up, interest in conjunction and disjunction fallacies, as well as violations of binary complementarity, is not merely theoretical. Probabilistic errors in applied domains—such as medicine, law, and public policy—can lead to significant real-world consequences, including diagnostic errors, misjudged legal probabilities, and flawed policy decisions (Bar-Hillel and Neter, 1993; Crupi et al., 2018; Guthrie et al., 2009; Wojciechowski and Pothos, 2018). Recognizing the prevalence and impact of these biases underscores the importance of understanding probabilistic reasoning not only in humans but also in emerging computational agents such as LLMs, which are increasingly consulted for judgment and decision-making tasks.

### 1.2 Probabilistic reasoning fallacies in LLMs

Emerging evidence indicates that LLMs may exhibit patterns of probabilistic judgment that partially parallel human cognition. Studies report that earlier models (e.g., GPT-3 and GPT-3.5) often commit conjunction or disjunction fallacies and sometimes underperform relative to human participants, suggesting bounded rational or heuristic-like reasoning (Binz and Schulz, 2023; Chen et al., 2023; Koralus and Wang-Maścianica, 2023; Suri et al., 2023; Wang et al., 2024; Yax et al., 2024). More advanced systems such as GPT-4 show fewer such errors and, in some cases, superhuman performance on structured probabilistic benchmarks (Hagendorff et al., 2023; Wang et al., 2024; Yax et al., 2024). However, it remains unclear whether LLMs’ successes reflect genuine probabilistic competence or the superficial avoidance of specific pitfalls. Moreover, prior work seldom distinguishes single from double violations or examines binary complementarity, limiting our understanding of the competence of such models on probabilistic tasks. The broader theoretical question—whether LLM biases arise in a way analogous to human-like heuristics, idiosyncratic architectural constraints, or training-data artifacts—remains unresolved (Macmillan-Scott and Musolesi, 2024).

### 1.3 Contribution of the present study

The present study systematically evaluates whether GPT-5 adheres to core probabilistic axioms or exhibits characteristic fallacies in its judgments. We focus on three foundational phenomena: conjunction fallacies, disjunction fallacies, and violations of binary complementarity. Extending previous research, we explicitly distinguish single and double violations, allowing us to probe deeper into the capabilities of such. We further incorporate complementarity, a central but understudied test of LLM probabilistic competence, motivated by recent findings that humans can violate this important constraint (Huang et al., 2025).

By comparing GPT-5’s responses to human behavioural data, we examine whether its deviations from classical probability theory replicate human patterns, partially overlap with them, or diverge in ways unique to language-based computation. Beyond measuring error prevalence, we analyse the structural profile of GPT-5’s misjudgement’s to determine whether its reasoning resembles bounded rationality, noise-driven inconsistency, or a qualitatively different form of probabilistic processing. By mapping the alignment or divergence between human and artificial reasoning, we provide a foundation for evaluating whether LLMs should be treated as probabilistically reliable advisers or as systems whose coherence requires continual scrutiny.

Put differently, the motivation for the present study ultimately concerns whether LLM (specifically GPT-5) judgments concerning probabilities can be trusted or not. This is a timely question, in light of the fact that LLMs are becoming increasingly popular as sources of advice (Bright et al., 2024; Hou et al., 2024; Jung et al., 2024) and recent calls for principled evaluation frameworks when LLMs are used as judgment or decision-support systems (Lee et al., 2026). As noted, quality and trustworthiness of probability judgments depend on two dimensions. The first dimension is whether probabilities are set in an accurate way, given the content of the premises. For example, a judgment that the probability it will rain in London in December is 10% is highly misleading. The second dimension – and the focus of the present work – is whether probabilities relate to each other in the correct way. For example, regardless of whether individual probabilities are estimated accurately or not, if an LLM decides that the probability that it will rain and snow in London in December is greater than just the probability it will rain, this is a (conjunction) fallacy and an error under most circumstances (Pothos et al., 2017).

Finally, the probabilistic task we employ concerns political judgments (Ceron et al., 2024; Santurkar et al., 2023). Therefore, it is useful to examine whether GPT-5 displays some sensitivity to modern politics. Otherwise, internal consistency of probabilistic assignment (or otherwise) might simply reflect non-committal probabilities, that is, probabilities which are fairly uniform across the relevant events. To this end, we conducted an assessment of GPT-5’s political sensitivity, by comparing its probability estimates for election outcomes across parallel, matched prompts involving the two candidates relevant to the probability judgments.

## 2 Models of probabilistic reasoning

A wide range of formal frameworks have been developed to account for systematic deviations from (baseline) classical probability theory in human judgment (Busemeyer and Bruza, 2012; Tversky and Kahneman, 1974, 1983; Zhu et al., 2020). Broadly, these approaches fall into three families: classical Bayesian models, which (in their baseline form) preserve the Kolmogorov axioms and attribute errors to cognitive limitations or noise, heuristic approaches, which explain fallacies with individual, often unconnected principles, and quantum probability models, which posit a fundamentally different geometric structure of mental representations that naturally gives rise to certain violations. In this work, we focus on Bayesian and quantum approaches, only because these approaches provide more constraints regarding what probabilistic inference should be like.

Classical Bayesian models treat probabilities as measures over subsets of a fixed sample space (Oaksford and Chater, 2007). Within this framework, conjunctions, disjunctions, and conditional probabilities must satisfy additivity and coherence constraints. To explain departures from these constraints, early classical accounts introduced stochastic mechanisms based on sampling and noise. In the probability-plus-noise model (Costello and Watts, 2016, 2014, 2018), individuals maintain internally coherent classical probabilities, but their expressed judgments are corrupted by “recording noise.” Increasing the noise for complex events allows the model to reproduce conjunction and disjunction fallacies. A related approach, the Bayesian sampler (Zhu et al., 2020), assumes that people generate probability estimates from finite memory samples governed by a symmetric beta prior. Smaller samples for conjunctions and disjunctions lead those judgments to be more heavily influenced by the prior, yielding systematic biases. Both models produce mostly similar predictions.

Quantum probability models offer a contrasting theoretical foundation for probabilistic inference, by representing probabilities as geometric projections of a cognitive state vector in a Hilbert space (Busemeyer and Bruza, 2012). A central insight of this framework is that certain cognitive questions are incompatible: forming a judgment about one event changes the internal state relevant for judging another (Busemeyer and Bruza, 2012, 2024; Pothos and Busemeyer, 2013, 2022). When events are incompatible, quantum theory requires conjunctions and disjunctions to be computed sequentially, rather than through classical set-theoretic rules. Busemeyer et al. (2011) applied this principle by proposing a “more-likely-first” rule: people evaluate the event they judge more probable first and then the one considered less likely. Under this assumption, the quantum model predicts directional fallacies—conjunction errors involving the less probable event and disjunction errors involving the more probable event—while strictly prohibiting double fallacies. For example, if P(A)>P(B) , the sequential rule requires P(Aand thenB)≤P(A) , making it impossible for the conjunction to exceed both constituents. Equivalent constraints govern disjunctions. Thus, quantum models explain certain systematic patterns in human judgments, while sharply restricting others.

Early quantum models left open how internal quantum probabilities translate into the noisy numerical ratings that participants report. Huang et al. (2025) addressed this limitation by introducing the quantum sequential sampler, a hybrid model that merges quantum state dynamics with sampling mechanisms inspired by classical memory-based accounts. The quantum sampler assumes that people draw sequential samples from a quantum cognitive state and convert these samples into explicit numerical judgments subject to estimation noise.

An innovation of the quantum sampler is its use of POVMs (positive operator-valued measures) to compute probabilities, instead of the ideal projective measurements assumed in earlier work (Busemeyer et al., 2011). POVMs allow a controlled mismatch between the underlying cognitive state and the observed response, providing a principled noise model that generalizes classical “recording noise” into the quantum framework. This increased flexibility enables the model to capture a broader spectrum of empirical phenomena, including systematic violations of binary complementarity and the presence of both double conjunction and double disjunction fallacies—patterns the earlier quantum models could not accommodate.

To evaluate the quantum sequential sampler against the predominant Bayesian model (the Bayesian sampler), Huang et al. (2025) conducted a large-scale study with 1,451 U. S. participants shortly before the 2020 US presidential election. Each participant provided 78 judgments, including marginal, conditional, conjunctive, disjunctive, and negation probabilities across three event pairs. Across multiple generalization tests, the quantum sampler consistently outperformed the Bayesian sampler, offering the best available joint explanation of the full pattern of human probabilistic judgments.

## 3 Experiment

Briefly, the main aim of this study is to examine whether GPT-5, a state-of-the-art LLM, exhibits human-like patterns of probabilistic bias when confronted with the same tasks used in recent behavioural studies, specifically Huang et al. (2025). This study asked U.S. participants to judge the likelihood that the 2020 presidential candidates—Joe Biden and Donald Trump—would win various combinations of states. Human responses reliably violated classical axioms, including complementarity, conjunction, and disjunction constraints.

Our central objective is to assess whether GPT-5, when instructed through persona-based prompts mirroring the demographic profiles of Huang et al.’s participants (age, gender, educational attainment, and geographic region), reproduces these non-normative patterns. The persona strategy enables a one-to-one mapping between individual human participants and corresponding model simulations, thereby allowing a fine-grained comparison of response distributions.

### 3.1 Materials and methods

#### 3.1.1 Human experimental paradigm

Huang et al. (2025) investigated probabilistic reasoning about the 2020 U.S. presidential election using a structured set of probability-judgment tasks. Each participant provided 78 probability judgments concerning whether either or both candidates would win specific states. The paradigm involved two triplets of states—Ohio–Missouri–Michigan (Triplet 1; T1) and Georgia–Montana–Nevada (Triplet 2; T2). For each triplet, participants evaluated all possible combinations of events, comprising six marginal events (e.g., Biden wins Ohio), 12 conjunctions (e.g., Biden wins Ohio and Missouri), 12 disjunctions (e.g., Trump wins Nevada or Biden wins Georgia), and 12 conditionals (e.g., Biden wins Michigan given Trump wins Missouri). Each composite event was additionally presented in the reversed order to counterbalance potential order effects, contributing 36 further items and bringing the total to 78.

To reduce anchoring from composite judgments, all marginal probabilities were elicited first. The remaining items were grouped into thematic blocks, and items within each block were randomized. Participants responded using a continuous slider from 0 to 100.

The original study employed a 2 × 2 between-subjects design varying State Triplet (T1 vs. T2) and Complexity (Low Complexity, LC, vs. High Complexity, HC). Complexity was manipulated by structuring composite-event blocks either by state pair (LC) or by intermixing different pairs within the same block (HC), ostensibly manipulating complexity (this is because the greater the range of events, the more complex the corresponding probability representations needed, from a Bayesian perspective). The four groups—T1LC, T1HC, T2LC, and T2HC—allowed the authors to test whether complexity modulated violations of probabilistic coherence.

For the present GPT-5 investigation, we use only the low-complexity conditions (T1LC and T2LC). Huang et al. reported no systematic effects attributable to complexity, and the LC conditions provide a cleaner, more interpretable structure for persona-driven simulations. These two groups included 284 and 269 participants, respectively, yielding 553 distinct demographic personas used in GPT-5 data collection.

#### 3.1.2 GPT-5 data-collection paradigm

Model-generated data were obtained using GPT-5-2025-08-07, a release from OpenAI’s GPT-5 reasoning family issued in August 2025. All data collection was conducted during November 2025 through the OpenAI Python API, using standardized scripts to ensure complete procedural uniformity and reproducibility across sessions. The overall objective was to replicate the structural, instructional, and cognitive conditions of the human experiment as faithfully as possible, while maintaining reasonable experimental control. Specifically, our intention was to capture GPT-5 reasoning ‘by default’, in the same way that participants in a typical psychology experiment (such as the one from Huang et al.) are invited to reason ‘by default’. In the human case, by default implies certain instructional manipulations and procedural limitations. We aimed to simulate analogous conditions in GPT-5, in a very approximate sense (given the lack of understanding in GPT-5 behaviour and the practical impossibility of systematically controlling across different instructional and procedural variants). We attempted to do this in three ways. First, by providing suitable instructions to GPT. Second, by asking GPT to simulate (very broadly speaking) some general characteristics of the participants. Finally, by ensuring that each judgment is carried out in a broad content of memory of previous judgments, which we considered analogous to that of humans.

Each simulated session began with a persona-initialization prompt embedding the demographic characteristics of a specific participant from the T1LC or T2LC human groups. For each human participant, four demographic variables were loaded from the study dataset—age, gender, education level, and U.S. state of residence—and inserted into a fixed natural-language template that generated the persona-initialization prompt. This prompt specified the participant’s demographic attributes in a single descriptive sentence (e.g., “You are a 54-year-old female with a bachelor’s degree living in Ohio”) and was followed by standardized task instructions reproduced verbatim from the human experiment. No additional psychological, behavioural, or personality descriptors were added; the personas differed only in the demographic information originally provided by each human participant. This procedure ensured a one-to-one correspondence between human participants and simulated personas and prevented any experimenter-induced variance in persona content.

The instructions presented to human participants were reproduced verbatim. These included the directive to give intuitive, first-impression estimates, to provide a single integer between 0 and 100 for each item, and to respond as if the 2020 election outcome were not yet known. GPT-5’s response to this instructional prompt remained in the message history throughout the session, maintaining a consistent cognitive frame across all 553 runs. Note that it is unclear whether GPT-5 can suppress knowledge like this, in a way analogous to how humans cannot suppress knowledge. In the Discussion we consider whether factual knowledge of the election results might impact on model behaviour.

Following initialization, GPT-5 received the complete set of 78 probability-judgment items arranged in the same block structure used in the human study. Within each block, item order was randomized independently for every persona, thereby mirroring the block-level randomization procedure applied to human participants. Each item was embedded in a concise prompt instructing the model to return a single numeral (0–100). Responses were parsed automatically and saved to structured output files.

One design element was the implementation of a bounded working-memory constraint, intended to approximate human cognitive limitations during sequential judgment tasks. Although GPT-5 normally operates with an extensive context window, note that the API we employed ‘resets’ the memory window with each query. We constrained the model to a sliding window consisting only of the original persona-initialization message and the seven most recent question–answer pairs. All earlier interactions were removed from the message history prior to issuing the next item. This procedure prevented the model from maintaining a perfect record of its earlier outputs and forced it to operate within a cognitively limited environment, more comparable to human working memory. The seven–pair limit was selected because it approximates the upper bound of human working-memory capacity for maintaining discrete verbal–numerical items during sequential judgment tasks, consistent with classic capacity estimates (Miller, 1956) and contemporary evidence that effective working memory in complex tasks is often substantially lower (Cowan, 2001). Recent work has emphasized that imposing such psychological constraints can reveal more human-like patterns of reasoning in LLMs (Hagendorff et al., 2023; Liu et al., 2025) and the present design follows this approach. Whether the length of ‘seven’ is the most appropriate memory constraint is less relevant: the point is that GPT-5 was asked to perform in a context in-between much more extensive memory of previous responses or no memory at all of previous responses.

All interactions with GPT-5 were conducted via the standard Chat Completions API. No browsing, web search, plugins, or external tool access were enabled; all probability judgments were therefore generated solely from the model’s pretrained internal knowledge and the conversational context. API calls included only the model identifier, message history, and the sampling temperature (set at 1.0), while all other parameters remained at their documented defaults. The *reasoning effort* parameter was not explicitly set; according to the OpenAI API reference, this parameter defaults to medium for gpt-5-2025-08-07 and is applied automatically when not specified (OpenAI, 2026). Accordingly, all sessions reflect the model’s standard, tool-free behaviour under typical usage conditions, rather than behaviour influenced by experimental tuning or enhanced reasoning controls.

Each persona generated an independent run, resulting in 553 sessions × 78 items per session = 43,134 model-generated probability estimates.

### 3.2 Results

A series of paired-samples *t*-tests compared the probabilistic reasoning performance of human participants with that of matched GPT-5 personas. Across all measures, GPT-5 demonstrated markedly stronger adherence to probabilistic principles and substantially lower rates of classical reasoning fallacies. We present the results in detail in the following sections.

*Binary complementarity*: following Huang et al. (2025), we defined P(A) as the judged probability that Donald Trump would win a given state and P(¬A) as the probability that Joe Biden would win that same state. Under the classical axiom of binary complementarity (Equation 3), these two probabilities should sum to unity. Accordingly, marginal violations of complementarity were quantified for each participant or GPT-5 persona as ∣P(A)+P(¬A)−1∣ , averaged across the three state triplets. To assess whether joint probability judgments respected the extended complementarity constraint (Equation 4), we computed ∣P(A∧B)+P(A∧¬B)+P(¬A∧B)+P(¬A∧¬B)−1∣ , averaged across six possible configurations. This yielded one marginal and one joint complementarity score per individual.

GPT-5’s complementarity performance was effectively normative. Marginal violations were extremely small (M = 0.00004, SD = 0.00045), approaching numerical rounding error. Humans showed much larger and more variable deviations (M = 0.248, SD = 0.238). Joint complementarity revealed an even stronger divergence: GPT-5’s deviations were minute (M = 0.00108, SD = 0.00820), whereas human judgments departed from the theoretical value by more than a full probability point on average (M = 1.250, SD = 0.656).

Paired-samples *t*-tests confirmed that GPT-5 personas adhered significantly more closely to complementarity than human participants for both marginal, *t*(552) = −24.53, *p* < 0.001, and joint probabilities *t*(552) = −44.74, *p* < 0.001.

**Figure 1:**
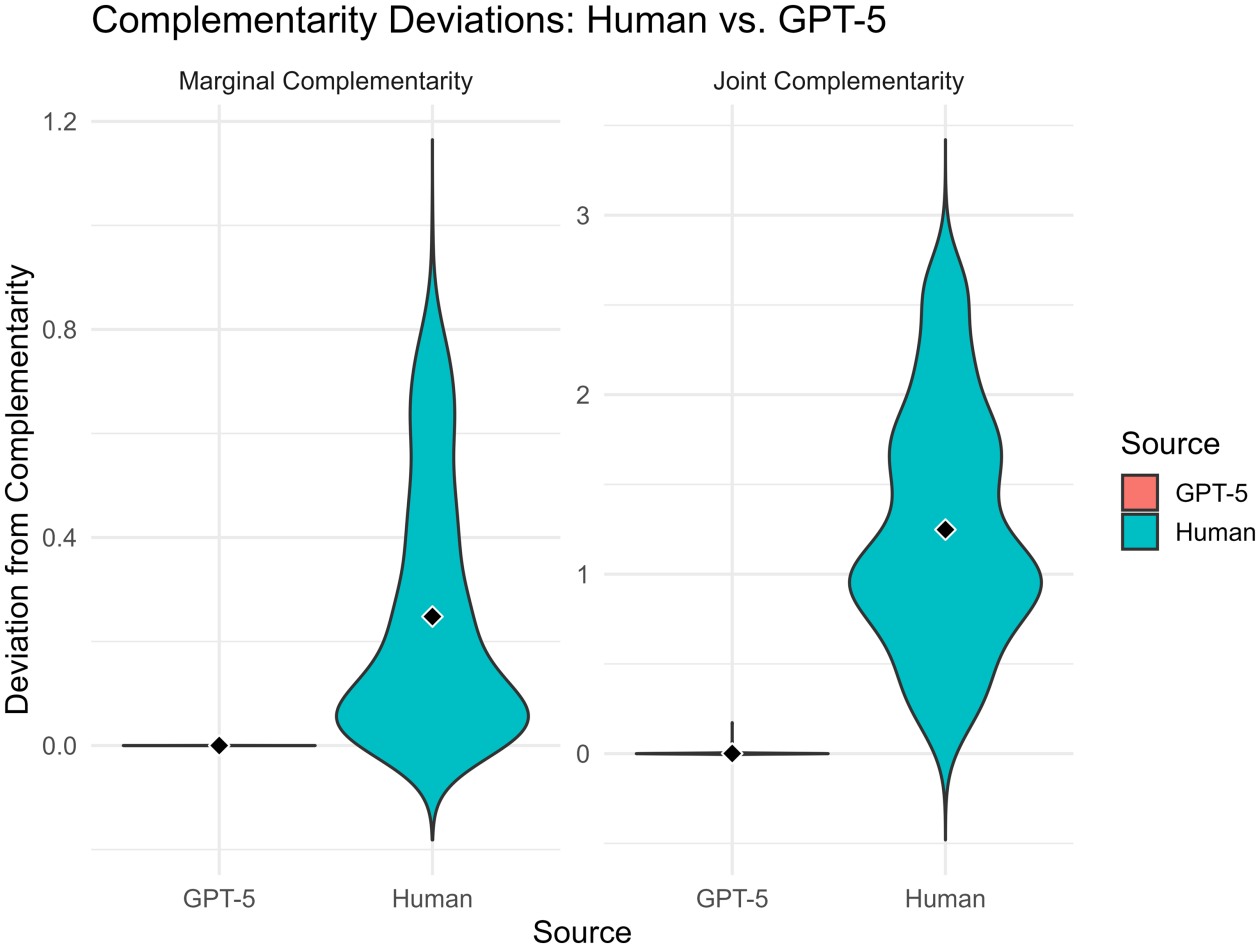
Violin plots depicting the distributions of deviations from marginal (left) and joint (right) complementarity for human participants and GPT-5 personas. Human responses display wide, variable departures from the complementarity constraint, with many participants exhibiting large violations. In contrast, GPT-5 personas show extremely narrow distributions centred near zero, indicating almost perfectly coherent probability judgments across both marginal and joint constraints. Regarding GPT-5 performance, in this case because of the narrowness of the distribution the ‘orange’ is invisible.

Figure 1 visualizes these patterns, illustrating the model’s systematic coherence compared with the considerable noise in human intuitive probability judgments.

*Conjunction fallacies*: to quantify violations of the conjunction rule, we computed a conjunction-fallacy score (CF) for each item involving events A and B. A conjunction fallacy occurs when the judged probability of the conjunction exceeds at least one constituent probability. For item *i*, the magnitude of this violation was computed as defined in Equation (5):


$$CF_{i}=\max\{P{\left(A\wedge B\right)}_{i}-P{\left(A\right)}_{i},0\}+\max\{P{\left(A\wedge B\right)}_{i}-P{\left(B\right)}_{i},0\}$$ (5)

We also derived a double conjunction fallacy (DCF) score, reflecting cases where the conjunction exceeds both constituents, as defined in Equation (6):


$$\mathrm{DCF}=\max\{P{\left(A\wedge B\right)}_{i}-\max[P{\left(A\right)}_{i},P{\left(B\right)}_{i}],0\}$$ (6)

Human participants showed a high prevalence of conjunction-rule violations: 58.5% of conjunction judgments contained at least one fallacy. Of these, 62.9% were single fallacies (CF > 0 but DCF = 0), and 37.0% were double conjunction fallacies (DCF > 0).

By contrast, GPT-5 displayed much more normative behaviour. Only 18.8% of model-generated judgments contained any conjunction fallacy and almost all were of the single-fallacy type (99.5% CF; 0.5% DCF). Thus, while the model occasionally overestimates a conjunction, it almost never commits the double fallacy.

Participant-level averages showed large differences. GPT-5 produced significantly fewer conjunction fallacies overall, *t*(552) = −24.79, *p* < 0.001, and dramatically fewer double conjunction fallacies, *t*(552) = −18.09, *p* < 0.001.

**Figure 2:**
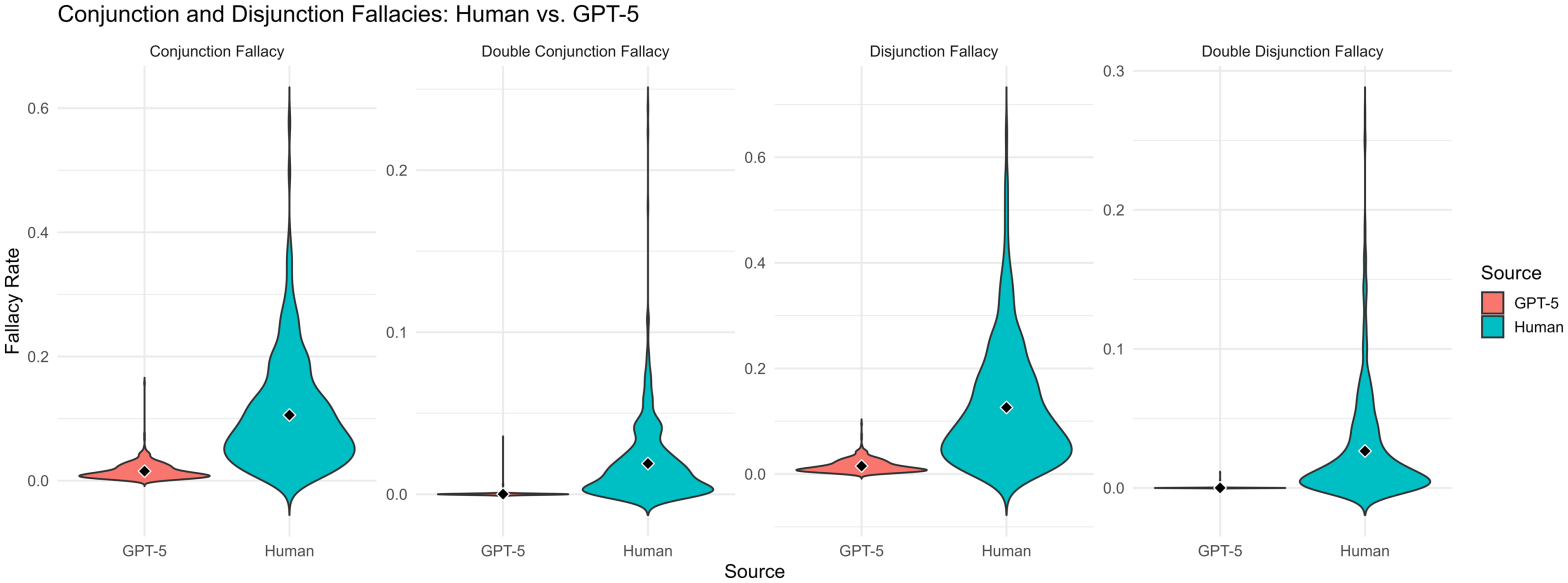
Distributions of conjunction and disjunction fallacy rates for human participants and GPT-5 personas. The left panel displays the distributions of single (CF) and double (DCF) conjunction fallacy rates, and the right panel displays the corresponding distributions for single (DF) and double (DDF) disjunction fallacies. Each violin plot reflects the participant-level mean fallacy score computed across all relevant items. Human participants exhibit broad, positively skewed distributions across all fallacy types, with substantial variability and frequent double-fallacy violations. In contrast, GPT-5 personas cluster tightly around zero, showing low but nonzero rates of single fallacies and near-zero rates of double fallacies. The marked difference in distributional shape highlights GPT-5’s substantially higher adherence to probabilistic coherence relative to human respondents.

As shown in Figure 2 (left panel), human CF and DCF distributions are broad and positively skewed, with substantial variability and frequent severe violations. GPT-5 personas cluster tightly near zero, exhibiting small but nonzero CF values and virtually no DCF.

*Disjunction fallacies*: an analogous procedure was used to quantify violations of the disjunction rule. For each item involving events A and B, a disjunction fallacy occurs when the judged probability of the disjunction is lower than one or both constituent probabilities, as defined in Equation (7):


$$DF_{i}=\max\{P{\left(A\right)}_{i}-P{\left(A\vee B\right)}_{i},0\}+\max\{P{\left(B\right)}_{i}-P{\left(A\vee B\right)}_{i},0\}$$ (7)

Double disjunction fallacies (DDF) occur when the disjunction is lower than both constituents, as defined in Equation (8):


$$\mathrm{DDF}=\max\{\min[P{\left(A\right)}_{i},P{\left(B\right)}_{i}]-P{\left(A\vee B\right)}_{i},0\}$$ (8)

Human participants again showed high violation rates: 57.5% f disjunction judgments contained at least one fallacy, of which 60.1% were single fallacies and 39.9% were double fallacies.

GPT-5 once more showed markedly more normative behaviour. Only 18.7% of disjunction judgments contained any violation and nearly all were single fallacies (99.5% DF; 0.5% DDF). Double disjunction fallacies were effectively absent.

As above, participant-level averages revealed significant human–GPT-5 differences. GPT-5 exhibited dramatically fewer disjunction fallacies, *t*(552) = −23.01, *p* < 0.001, and substantially fewer double disjunction fallacies, *t*(552) = −16.57, *p* < 0.001.

Figure 2 (right panel) shows the same qualitative pattern as for conjunction fallacies: human distributions are wide and positively skewed, whereas GPT-5 distributions are tightly concentrated near zero.

*Correlation between conjunction and disjunction fallacy rates*: It is informative to examine whether susceptibility to conjunction and disjunction fallacies covaries. Because both constitute violations of normative probability theory, one might expect a positive association: individuals prone to committing conjunction fallacies may also be prone to committing disjunction fallacies. To assess this possibility, we computed Pearson correlations between mean CF and DF rates within each group.

Among human participants, the correlation was small and not statistically significant, *r*(551) = 0.03, *p* = 0.496 indicating that susceptibility to the two fallacies was largely independent. In contrast, for GPT-5 personas, the correlation was extremely strong and positive, *r*(551) = 0.97, *p* < 0.001, indicating a near-perfect coupling between the two types of fallacious judgments.

This dissociation suggests that, whereas humans exhibit largely independent tendencies to commit conjunction versus disjunction fallacies, GPT-5’s non-normative probability judgments—when they occur—are tightly linked. Such coupling is consistent with the presence of a single underlying generative mechanism driving the model’s residual violations of normative probability theory, rather than distinct cognitive processes for each fallacy type.

Note, the quantum probability model proposed by Busemeyer et al. (2011) offers a principled account of why conjunction and disjunction fallacies may covary. In this framework, probability judgments are subject to order effects, such that the judged likelihood of a conjunction depends on the sequence in which constituent events are mentally processed. Processing a likely event first increases the availability of thoughts supportive of a subsequent unlikely event, thereby inflating the perceived probability of the conjunction. Conversely, processing the unlikely event first reduces the availability of supportive thoughts, lowering the conjunction probability. These order-dependent dynamics induce a systematic relationship between conjunction and disjunction judgments, providing a unified explanation for the co-occurrence of both fallacies observed in human reasoning. However, in the subsequent quantum model of Huang et al. (2025), the additional noise in responses makes a prediction of correlation between conjunction and disjunction fallacies less straightforward.

**Figure 3:**
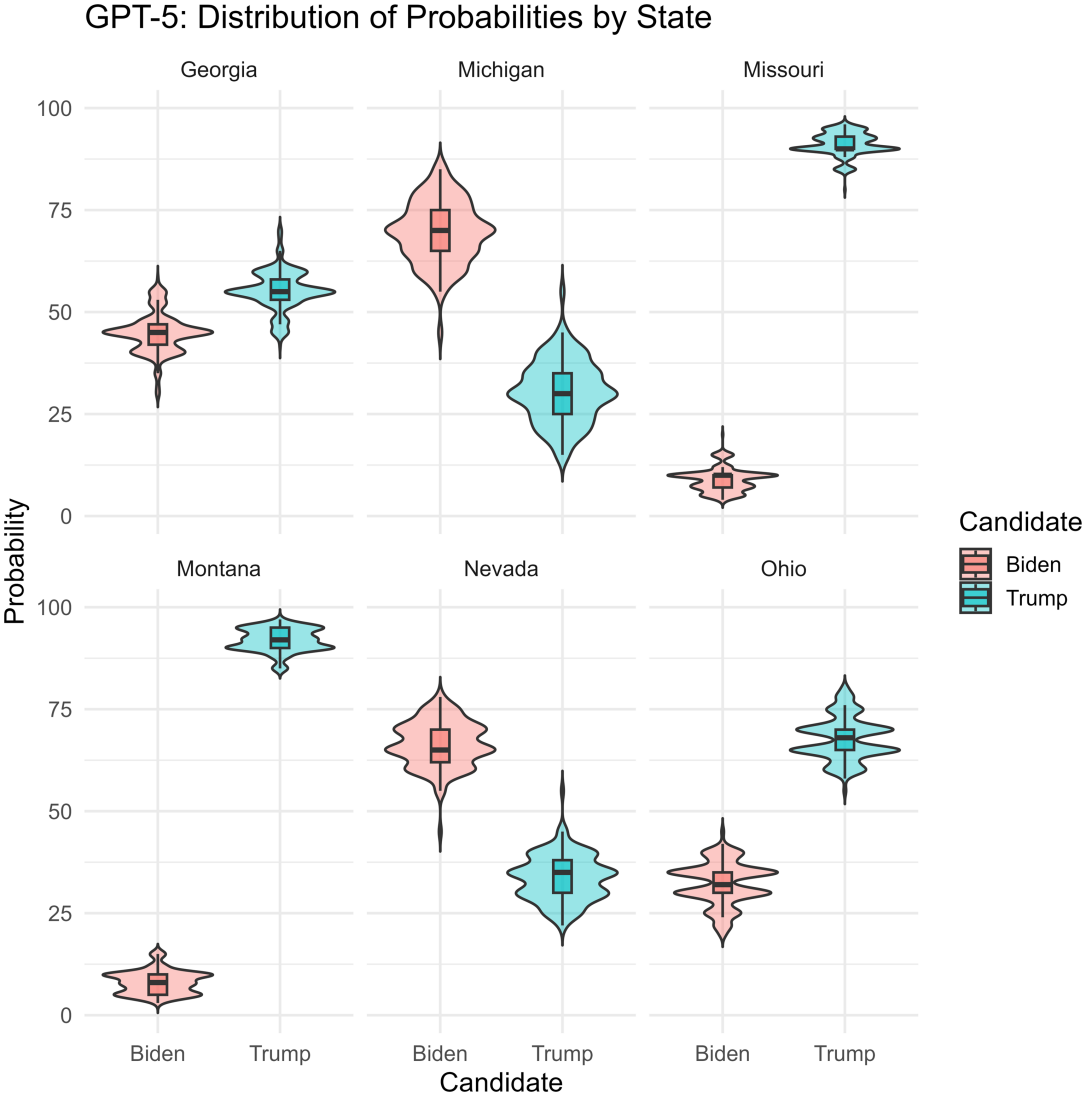
Distribution of GPT-5’s probability estimates for Biden and Trump across six U.S. states. Violin plots depict the distribution of GPT-5’s repeated probability estimates for each candidate–state pairing, with embedded boxplots indicating the median and interquartile range. The figure highlights substantial, state-dependent variation in GPT-5’s win-probability predictions.

*Model-level political sensitivity*: to examine whether GPT-5 displays some political sensitivity, we analysed its probability estimates for Democratic and Republican candidates across multiple U.S. states. Figure 3 displays the distribution of GPT-5’s probability estimates for Biden and Trump winning each of six states, based on prompts of marginal events (e.g., “What is the likelihood that Biden will win Ohio?”). Visual inspection readily reveals systematic variation in the model’s probability assignments as a function of both state and candidate. Note, Huang et al. (2025) did not collect corresponding results for humans (the assumption in that study being that naïve participants would have some political sensitivity).

To formally assess these differences, we conducted paired-samples *t*-tests comparing GPT-5’s probability estimates for Trump versus Biden within each state. Results are reported in Table 1. Positive mean differences indicate higher probabilities assigned to Trump, whereas negative values indicate higher probabilities assigned to Biden. GPT-5 assigned significantly higher probabilities to Trump in Missouri, Ohio, Georgia, and Montana (all *p* < 0.001), while significantly higher probabilities were assigned to Biden in Michigan and Nevada (all *p* < 0.001). Mean differences ranged from −39.13 to 83.83, indicating substantial candidate-specific modulation in the model’s probability estimates across states.

**Table 1:** Paired-samples *t*-test results comparing GPT-5’s probability estimates for Trump versus Biden across six U.S. states.

State	Mean difference (Trump–Biden)	*t* (DF)	*p*-value
Michigan	−39.13	−43.71 (283)	<0.001
Missouri	82.31	248.49 (283)	<0.001
Ohio	35.56	56.70 (283)	<0.001
Georgia	10.48	17.81 (268)	<0.001
Montana	83.83	247.53 (268)	<0.001
Nevada	−32.91	−48.78 (268)	<0.001

Positive values indicate higher probabilities assigned to Trump; negative values indicate higher probabilities assigned to Biden.

Taken together, these results indicate that GPT-5 does not treat these electoral possibilities as equivalent. Instead, its probability estimates exhibit systematic, state-dependent asymmetries that reflect political sensitivity.

## 4 Discussion

The present findings reveal a clear and systematic divergence between human and GPT-5 probabilistic reasoning. Human participants displayed the well-documented pattern of conjunction and disjunction fallacies, including a substantial number of double violations (Crupi et al., 2018; Tversky and Kahneman, 1983; Wojciechowski and Pothos, 2018). These errors were accompanied by large and heterogeneous violations of both marginal and joint complementarity (Huang et al., 2025). In some cases, such errors exceeded theoretical limits by wide margins. This broad variability is consistent across behavioural studies and indicates a plurality of generative mechanisms for probability judgments. Possible mechanisms include Bayesian and quantum processes, but perhaps heuristics too, which are outside formal probabilistic models (Huang et al., 2025).

GPT-5, by contrast, showed a markedly different profile. The model produced fewer single fallacies and almost no double fallacies; its complementarity deviations were extremely small—near the level of numerical noise. This narrow spread of GPT-5 results indicates that the model’s rare departures from coherence do not arise from noisy representations or fluctuating uncertainty. Instead, they reflect a highly stable and internally constrained inferential process.

The correlation analysis further highlights this structural distinction. Among human participants, conjunction and disjunction fallacies were essentially uncorrelated, this suggests that these violations originate from processes which are not restricted to the ones assumed in formal probabilistic models (as noted, noise-less quantum theory predicts a correlation between CFs and DFs). GPT-5 displayed the opposite pattern: when errors occurred, they were strongly correlated across CFs and DFs. This tight coupling implies a common mechanism in the workings of the model, which is the cause of both kinds of fallacies.

Specifically, when GPT-5 does produce fallacies, results show that its behaviour is consistent with early quantum-probabilistic models, for example, the original model for CFs and DFs proposed by Busemeyer et al. (2011). This model allows single conjunction or disjunction fallacies, while strictly prohibiting double fallacies and enforcing complementarity. Moreover, in this model CFs and DFs are correlated with each other. In contrast, the quantum model proposed by Huang et al. (2025) could generate a broad range of violations—including frequent double fallacies and complementarity failures. The greater expressivity in Huang et al.’s (2025) models was due to the introduction of noise through POVMs and sample-based estimation. This additional flexibility does not match GPT-5’s profile. Overall, the results indicate that GPT-5 is not simply “less noisy” than humans. Its pattern of errors is qualitatively different. The model maintains a high degree of probabilistic coherence and, when it fails, it does so in a structured and internally consistent manner.

These differences suggest that GPT-5 relies on a computational mechanism for probabilistic assessment that is not isomorphic to the one employed in human cognition, even under task conditions designed to approximate human working-memory constraints. It is possible that such a mechanism is just closer to normative requirement. A noteworthy alternative possibility is that GPT-5 attains better normative behaviour because, across iterations, it avoids inconsistencies due to random variation (e.g., compare with a human judge, who might be more lenient on some days because they are in a good mood, cf. Kahneman et al., 2021). Yet another possibility is that the high correlation between the CF and DF rates which is the main source of evidence for concluding that GPT-5 behaves more like the simple quantum model of Busemeyer et al. (2011) is due to the floor effects fallacies in GPT-5. Unfortunately, without identifying some situation for which GPT-5 does have high CF and DF rates, we cannot further examine this interesting issue. Finally, our analysis of political-content prompts indicates that GPT-5’s probability assignments are informed by a reasonable degree of political sensitivity. The model’s forecasts diverge systematically across states and candidates in election scenarios. This demonstrates that its probabilistic behaviour is not uniform across structurally similar prompts.

Having said all the above, there are several limitations. The most important one concerns the research question: our objective was to capture performance of GPT-5 “by default.” The rationale is that typical GPT-5 users would be interested in whether GPT-5 conducts probabilistic inference in a way analogous to that of humans or not, in typical use. The difficulty lies in that by default for humans often implies a particular context, including a mindset, information, processing constraints etc. We contend that the suitable sense of by default in GPT-5 has to include similar characteristics, that is, characteristics which would plausibly get GPT-5 to approach these probabilistic questions in a way similar to that of humans—the persona and memory manipulations were intended to capture some aspects of this.

How much did these manipulations affect results? It is difficult to have confidence of their validity. In the case of the persona matching, it has to be noted that, without this manipulation, the GPT-5 simulations would have been simpler–this is because, simply, persona matching required unique GPT-5 runs matched to individual participants. We investigated the rate of conjunction and disjunction fallacies separately, with an ANCOVA model with state triplet (there were two state triplets), gender, and education level (four levels: high school or less, some college, bachelor’s degree, postgraduate education) as between-participants independent variables and age as a covariate. State Triplet exerted a strong effect on both disjunction and conjunction fallacy rates (both *p* < 0.001). In contrast, no robust main effects of demographic persona variables were observed for either fallacy type (all *p* > 0.13, this includes the covariate, age). A modest interaction between gender and education emerged for disjunction fallacies (*p* = 0.041) and marginally for conjunction fallacies (*p* = 0.091). Overall, the evidence that persona matching had a measurable impact on GPT-5 behaviour is weak at best, though not completely negligible.

Regarding the memory window, our motivation for the particular length we used was that humans going through a similar task would be making their judgments in the context of other temporally proximal responses. For GPT-5, a choice has to be made regarding the extent of context of previous responses, given that the default setting of having each response given as if the GPT-5 mental state is ‘tabula rasa’ seems unrealistic. This was our thinking for setting a memory window of a particular size (cf. Hagendorff et al., 2023; Liu et al., 2025). Note, one way in which memory window might affect probabilistic coherence is if memory of earlier responses helps ensure consistency with later ones. Indeed, in early experimental work on probabilistic fallacies, the relevant questions would be typically presented together or in successive trials (e.g., Tversky and Kahneman, 1983). However, in more recent investigations (such as Huang et al., 2025), it cannot be assumed that relevant probability responses would be judged together, even with a lengthy working memory window. In general, we would argue that the claim of probabilistic coherence is independent of memory processes and rather about whether probabilities are *generated* in a coherent way. From this perspective, manipulations of memory window would be expected to just have minor contextual influences on responses (for human or artificial agents). While we think this approach is plausible given what we know, we did not systematically evaluate the importance of the memory window length and any conclusions here are conditional on this methodological choice.

Another potential limitation is that the study was carried out well after the 2020 US Presidential election. Therefore, the outcome of the election (including performance of the two candidates in different primaries) has been public knowledge that would be, presumably, accessible to GPT-5. With human participants, there is some evidence that, for fact-based propositions, probabilistic inference avoids common probabilistic fallacies (Collins et al., 2024; Karvetski et al., 2013). Whether this effect might have an analogue in GPT-5 is hard to assess. Nevertheless, empirically, there is a straightforward way to address this possibility. We ran a control whereby the task was framed as a hypothetical future election (the 2028 US Presidential election, which is the next scheduled such election), with as-yet to be identified candidates (instead of Biden and Trump in the original study, the probability questions were framed in terms of the Democratic and Republican candidates). For the purposes of this simulation, we randomly sampled 100 personas from the original set (50 for each triplet of states).

Contrary to expectation, there was an *even lower* rate for both CFs and DFs in the 2028 task vs. the 2020 one (respectively, Welch’s *t*(134.24) = 4.16, *p* < 0.001and *t*(160.26) = 4.74, *p* < 0.001). There were only 12.9% CFs and 14.7%DFs in the 2028 task. There were no differences between the two tasks regarding double CFs, double DF, and violations of binary complementarity (all *p*’s > 0.16). Therefore, we can certainly dismiss the possibility that low fallacy rates in 2020 version of the task were driven by post-election factual knowledge. There are many possible reasons for the difference in GPT-5 performance between the 2020 and 2028 task versions, including the fact that in the 2020 version the candidates were specific individuals. Rather than speculate on these differences, we highlight the main conclusion, which is that when testing GPT-5 with the version of the task best aligned with what human participants received, GPT-5 behaviour was closer to normative expectation.

More generally, it is unclear how impactful instructional and procedural manipulations are for GPT-5. In other work of ours, systematically exploring prompt engineering manipulations has had little effect. Likewise, in this case, we think it is likely that the impact of the instructional manipulations we adopted is, at best, minor. While it is tempting to systematically evaluate different variants regarding instructions and procedure, it is impractical to do so and, arguably, of less interest. These considerations are analogous to human experimental work: experimenters often devote considerable effort to fine-tuning instructions and procedures (e.g., randomisation protocols) to very exacting specifications. However, invariably, minor methods differences do not impact behaviour.

Regarding the present results, we believe the above issues are somewhat moot. The logic is this: by getting GPT-5 to behave more like humans (by simulating some characteristics of the participants, having memory limitations etc.), one could argue that we are making it more likely to observe fallacies in GPT-5 (since fallacies are an aspect of human behaviour). However, GPT-5 uniformly produced fewer probabilistic fallacies, compared to humans. That is, we can say that, *conservatively,* GPT-5 conducts probabilistic inference in a way more rational compared to humans.

## 5 Concluding comments

This study sought to determine whether GPT-5’s violations of probabilistic axioms resemble those exhibited by human reasoners or whether they follow a distinct structural pattern. Despite efforts to place GPT-5 in conditions approximating human constraints, the model’s behaviour diverged sharply from human patterns. The findings therefore do not support the view that GPT-5 simply approximates “more accurate humans.” Instead, GPT-5 appears to instantiate a coherent form of probabilistic inference, that resembles a noise-free quantum model rather than the psychologically noisier mechanisms underlying human probability judgments. This distinction is important for interpreting LLM outputs. GPT-5 can provide probability assessments that are, in general, internally consistent and formally well-structured, as far as we can tell from this study.

These differences carry practical implications. As LLMs increasingly serve in advisory roles—educational, professional, and personal—results regarding their consistently coherent but cognitively non-human probabilistic guidance may influence when users are likely to resort to LLMs (and for what kind of judgments) and the trust they place in LLM output.

Future work should examine whether GPT-5’s coherence extends to more complex probabilistic domains, including conditional inference, belief updating, and sequential decision processes. An open question is whether aligning LLM behaviour with human fallacy patterns is desirable—or whether the value of such models lies precisely in their departure from human probabilistic limitations. The present study provides an initial foundation of results, showing that GPT-5 represents a distinct and unusually normative mode of probabilistic reasoning that stands apart from both classical human errors and models designed to explain them.

## Data Availability

Publicly available datasets were analyzed in this study. This data can be found at: the data is in the study of Huang et al. (2025).
